# “Image to patient” equal-resolution surface registration supported by a surface scanner: analysis of algorithm efficiency for computer-aided surgery

**DOI:** 10.1007/s11548-022-02704-1

**Published:** 2022-07-13

**Authors:** Ewelina Świątek-Najwer, Marcin Majak, Michał Popek, Magdalena Żuk

**Affiliations:** Department of Mechanics, Materials and Biomedical Engineering, Faculty of Mechanical Engineering, Wrocław University of Science and Technology, Wrocław, Poland

**Keywords:** Computer-aided surgery, Image to patient registration, Surface registration, Aspect of resolution influence on registration

## Abstract

**Purpose:**

The “image to patient” registration procedure is crucial for the accuracy of surgical instrument tracking relative to the medical image while computer-aided surgery. The main aim of this work was to create an equal-resolution surface registration algorithm (ERSR) and analyze its efficiency.

**Methods:**

The ERSR algorithm provides two datasets with equal, high resolution and approximately corresponding points. The registered sets are obtained by projection of a user-designed rectangle(s)-shaped uniform clouds of points on DICOM and surface scanner datasets. The tests of the algorithm were performed on a phantom with titanium microscrews. We analyzed the influence of DICOM resolution on the effect of the ERSR algorithm and compared the ERSR to standard paired-points landmark transform registration. The methods of analysis were Target Registration Error, distance maps, and their histogram evaluation.

**Results:**

The mean TRE in case of ERSR equaled 0.8 ± 0.3 mm (resolution A), 0.8 ± 0.5 mm (resolution B), and 1.0 ± 0.7 mm (resolution C). The mean values were at least 0.4 mm lower than in the case of landmark transform registration. The distance maps between the model achieved from the scanner and the CT-based model were analyzed by histogram. The frequency of the first bin in a histogram of the distance map for ERSR was about 0.6 for all three resolutions of DICOM dataset and three times higher than in the case of landmark transform registration. The results were statistically analyzed using the Wilcoxon signed-rank test (alpha = 0.05).

**Conclusion:**

The tests proved a statistically significant higher efficiency of equal resolution surface registration related to the landmark transform algorithm. It was proven that the lower resolution of the CT DICOM dataset did not degrade the efficiency of the ERSR algorithm. We observed a significantly lower response to decreased resolution than in the case of paired-points landmark transform registration.

## Introduction

The “image to patient” registration procedure is one of the most crucial factors for efficient computer-aided surgery [1, 2]. The algorithm enables tracking of the surgical tool’s position on the image background to provide safety of vital structures and precise implementation of surgical scenarios.

The most intuitive and simple approach is paired-points matching of palpated on a patient points and corresponding points on the DICOM dataset [3, 4]. To increase the accuracy of palpation, adhesive markers [5] or bone-anchored invasive markers are scanned with patients [6]. The latter type of markers appears to be optimal, since the skin attachable surface markers may slip or detach. Unfortunately, bone-anchored markers require an invasive procedure before CT scanning. As for recognition in an image, the system often automatically recognizes the markers on the DICOM projections as a geometric pattern or center of gravity [6]. The essential limitation of paired-points approach is that the markers must stay still until palpation. However, there are many possible human movements involved by breathing, swelling, other tissue shifting or, in the worst case, the markers might be removed [6]. Hence, the superficial landmarks particularly prone to dislocation must be cautiously used. Another important aspect of accurate paired-points registration is a selection of points for calculations. A well-known algorithmic limitation is that fiducials cannot be collinear, but the spread of points also influences the result of registration. The calculated matrix might be adjusted just for a restricted region. Therefore, the user needs to be careful while planning the distribution of registration points to ensure uniform coverage of the operating field.

Surface matching is rarely applied in commercial computer-aided surgery systems. The method finds a registration matrix for pre- and intraoperative dataset without clarified correspondences between particular points [7, 8]. Intraoperative data are recorded using a small laser scanner or tracked pointer (such as z-touch by BrainLAB), laser or other scanners providing more data [6, 9], or even A-mode ultrasound transducer [10]. Typically, the number of registered points is hundreds or thousands [6]. In most cases, the applied algorithm is Iterative Closest Point (ICP), which iteratively regulates the registration matrix to reduce the distance error to acceptable or up to the maximal number of iterations. The method is stable and robust; however, it tends to lead to an improper result caused by stucking in a local extreme [11].

Usually, that procedure is preceded with paired-points registration so that surface matching does not get stuck in a local extreme [12]. Therefore, at least three (or four, depending on developed algorithm application) pairs of corresponding points measured in the two datasets are required.

The registration procedure also concerns various imaging datasets to obtain a fused model as a result, in the simplest case to evaluate landmark positions [13]. That procedure can also be performed for anatomical data without any physical markers [6]. A rarely raised issue is related to the digital models before registration. CT-based bone segmentation often creates a model with outer and inner surfaces. If the registration matrix is calculated for both outer and inner points of the surface, it is often observed that after the last ICP iteration for the stop condition (e.g., the maximal number of ICP iterations or acceptable error value), the two registered datasets may be glued but as mirror images [12]. To eliminate this undesirable effect, all models should be preprocessed to obtain the outer surface.

The number of works presents the surface matching method on two sets of similar points resolution. Yoo et al. noticed the importance of optimal image points cloud application corresponding to the intraoperative points cloud during surface registration [14]. Dong et al. analyzed the influence of scanner resolution on registration and proposed digitalization of various regions of the head to increase the accuracy [15]. The difference in resolution may lead to imprecise matching using the iterative Closest Point algorithm. The contribution of this work is the evaluation of CT image dataset’s resolution influence on the registration result while using data from high-resolution surface scanners and low-resolution DICOM data. Created projection algorithm provides the ability to increase and synchronize resolutions of both datasets and ensure the correspondence of points.

To evaluate the registration process quantitatively three definitions of errors are applied [2]: fiducial localization error (FLE) (errors in fiducial points), fiducial registration error (FRE) measured for fiducial points after registration, and target registration error (TRE) measured for points excluding fiducial points after registration. The errors are calculated as a root-mean-square distance error between the point and the corresponding point in the other dataset after registration transformation. Although the value is an objective evaluation, it still describes only a particular point or an averaged error calculated for certain points from the considered area. The authors rarely decide to evaluate the distribution of TRE mapped on the 3D model [10, 16] which in our opinion helps to visualize the efficiency of registration. In our work, we focus on target registration errors after surface registration and maps of accuracy.

Hoffmann et al. reported an average TRE of surface registration up to 2.1 mm using a 3D laser scanner from BrainLAB Vector Vision [17]. Lübbers et al. measured the error in the different head areas and the average error for Z-touch surface registration equaled 1.0 mm–1.2 mm depending on the area [16], while the number of registered points was 200. Lee et al. achieved TRE of surface registration equaling 1.0 mm [18]. Widmann et al. reported TRE of surface registration in the range of 0.8 mm up to even 4.9 mm [2], whereas the best results for surface registration were obtained for scanning using a laser scanner with up to 300 000 points in the face/maxilla area. In a study by de Boutray et al., the mean TRE of fibula surface registration preceded by robust initial registration equaled 0.8 mm [9]. Diakov obtained sub-millimeter registration error in the clinically relevant anatomical areas on the anterior skull and up to 1.5 mm for the entire head using vector field analysis for the surface registration [10]. Grauvogel et al. compared navigation accuracy after surface registration and after LED-mask registration (0.9 mm versus 0.8 mm) proving slightly higher efficiency of surface registration, especially in a skull base area [8]. Comparing the results with dates of publications, it appears that the TRE of surface registration techniques is nowadays in the range of less than a millimeter up to 1.5 mm. Despite this, we still observe intensive studies on the subject of registration accuracy by applying non-contact techniques and sophisticated mathematical approaches which could optimize that procedure as free of human errors introduced by palpation.

The paper is constructed as follows: the first section presents the aim of our study, the second section (Material and Method) describes the data used in our study, the algorithm of registration and methods of analysis of achieved results. Result section presents obtained outcomes in the form of tables, graphs, and 3D maps. The final section is a discussion of the results and plans for further improvements of the algorithm.

## Aim of study

The paper describes an image to patient equal-resolution surface registration algorithm. It applies the following data: DICOM CT scans and model achieved by a surface scanner (Artec Spider 3D scanner). The developed innovative algorithm combines two approaches: paired-points and surface registration on DICOM and Artec scanner datasets with equal (and higher than DICOM’s) resolution provided by a projection of the high-resolution point cloud in a rectangle shape (HRPR) on the two registered datasets. The location and size of the rectangle are selected by the user. To increase the registration accuracy in the full operation area, the user can locate in various regions a few high-resolution projection rectangles (HRPR). In the last stage of registration (to transform the DICOM and Artec scanner dataset to the dynamic reference frame DRF), the algorithm applies data recorded by palpation with a trackable pointer. A detailed description of the algorithm is presented in the Material and Method section.

The motivation for our work was to analyze the following issues:

1. a comparison of paired-points landmark transform registration (**LTR**) and equal-resolution surface registration algorithm (**ERSR**),
2. an analysis of preoperative CT imaging resolution influence on the map of distance between registered CT and Artec scanner datasets for both algorithms: **LTR** and **ERSR**.

## Material and method

The investigation was carried out using the MentorEye system created at Wrocław University of Science and Technology for planning and aiding oncologic surgery. The system works with Optical Tracking System Polaris Spectra from Northern Digital Incorporation to track the position of surgical tools and fulfill planned surgical scenarios. For planning and aiding surgery, the system applies image DICOM data (e.g., computed tomography).

The MentorEye system provides the possibility for image-patient registration by applying **ERSR**—equal-resolution surface registration algorithm—preceded by paired-points registration using landmark transform (**LTR**). The **ERSR** works for the three types of data: DICOM data, digital models achieved from 3D surface Artec 3D Space Spider scanner with a resolution up to 0.1 mm and accuracy of 0.05 mm, and data recorded while palpating by a trackable pointer (with Polaris Spectra tracking system, Northern Digital Inc., Canada, accuracy 0.25 mm). Characteristics of DICOM and scanner dataset are presented in Table 1.

**Table 1:** Characteristics of 3D Artec Space Spider scanner and DICOM CT datasets

	Characteristics
3D Artec Space Spider Scanner dataset (scanned facial part without mandible)	number of vertices in the model: 178,257
DICOM dataset	512 × 512 pixels, field of view 201 mm, slice thickness: 1.5 mm, resolution 0.39 mm/pixels

**Fig. 1:**
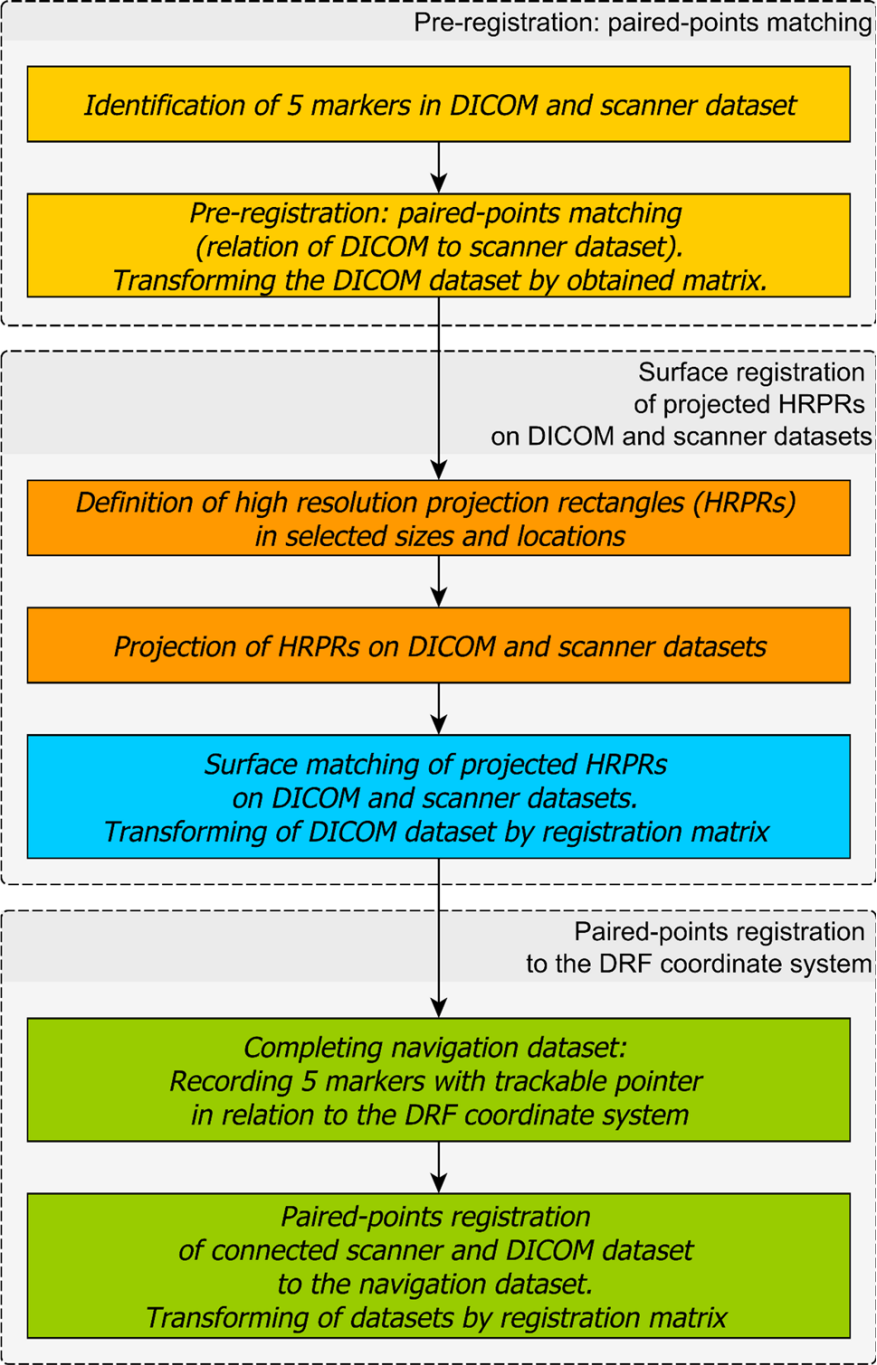
Flowchart of ERSR algorithm

**Fig. 2:**
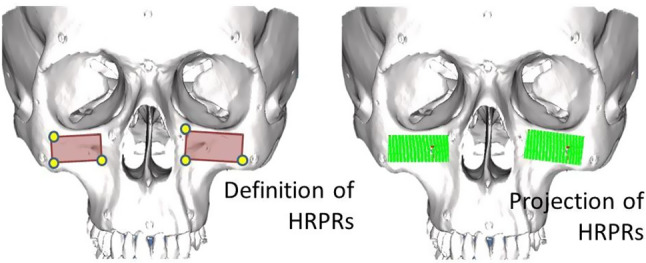
Definition of high-resolution projection rectangles (HRPRs) and projection of cloud on model surface

**Fig. 3:**
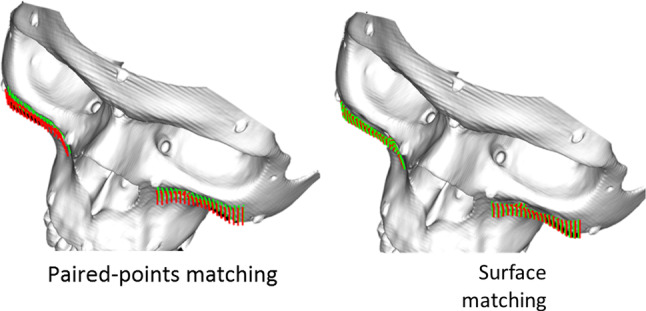
Result of paired-points matching (on the left side) and its correction by surface matching (on the right side)

**Fig. 4:**
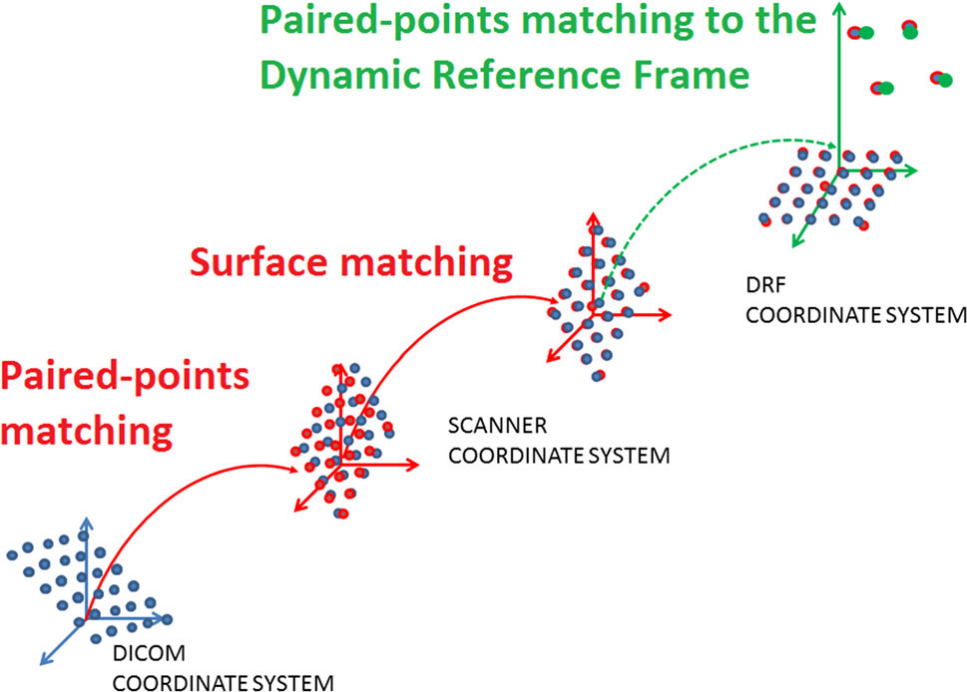
ERSR algorithm—transformations scheme

**Fig. 5:**
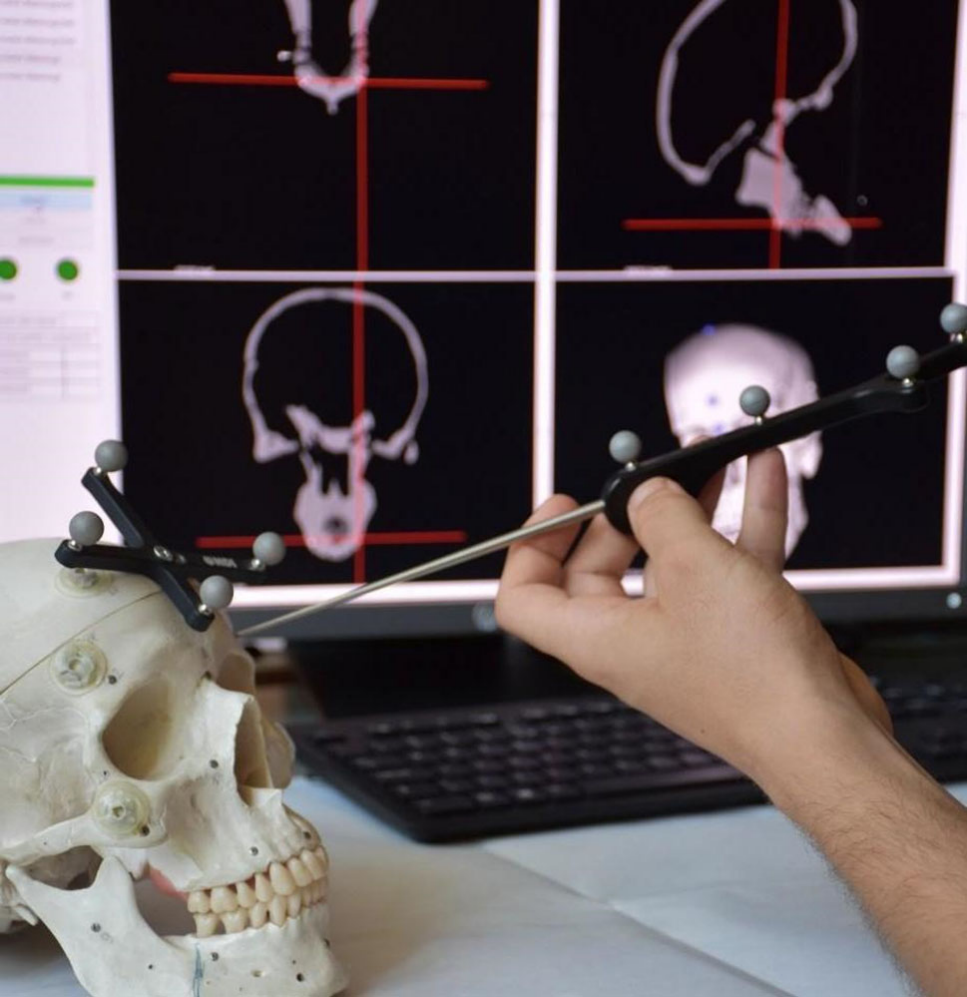
Palpating positions of 5 titanium landmarks by a trackable pointer

**ERSR** algorithm is presented on a flowchart in Fig. 1 and consists of the following stages:

1. The algorithm starts with paired-points matching of DICOM and 3D Artec Space Spider scanner dataset. This stage is also called landmark transform. The user identifies pairs of points (markers) on the outer surface of bone in the DICOM image dataset and on the model received by the 3D Artec Space Spider surface scanner.
2. The second stage is surface registration. It starts with operation helping to increase the DICOM dataset’s low resolution. The user defines a rectangle by pointing three points on a surface model. The corner points of the rectangle are pointed clockwise. According to the normal vector, the plane is translated by a distance d from the model surface. On the limited plane, a dense and uniform points cloud (HRPR) is defined to be projected on DICOM and 3D Artec Space Spider scanner datasets (as shown in Fig. 2). For each point of the cloud, a projection onto the model surface is performed in a direction opposite to the normal vector of the plane. If the projecting vector intersects the model more than once (i.e., model has outer and inner surface), only the superficial point with a lower value of z coordinate is selected. In case the operating area covers a few regions, it is possible to define several HRPRs. Projection of dense rectangles on pre-registered DICOM and scanner dataset provides equal, higher resolution than in case of DICOM dataset and better correspondence of points (Fig. 3). Surface matching is performed for the projected rectangle clouds of points on DICOM and scanner datasets (Fig. 4).
3. Registered in steps 1 and 2 and connected DICOM and scanner dataset can be used in computer-aided surgery after the landmark transform registration to the dynamic reference frame (**DRF**) coordinate system applying data recorded with a navigated pointer (Fig. 5).

The complete idea of coordinate transformations is presented on a graph shown in Fig. 4.

The implemented algorithms were tested on a skull phantom equipped with a dynamic reference frame (**DRF**) with optical markers. Titanium microscrews (with a diameter 1.0 mm and length 4.0 mm) were inserted into a plastic skull model (type: A20, 3B Scientific GmbH, Hamburg, Germany) as fiducial markers. The skull model was examined with a computed tomography (CT) scanner (Siemens Sensation Open) with 512 × 512 pixel dataset acquired at 1.5-mm slice thickness and resolution of 0.39 mm/pixels. Being aware that the lower slice thickness would be preferable to increase resolution and accuracy, we decided to apply that thickness (1.5 mm) as potentially used for scanning a patient in order to reflect a real approach in the clinical conditions. The images in Digital Imaging and Communication in Medicine (DICOM) format were sent to the registration module of the MentorEye system.

**Fig. 6:**
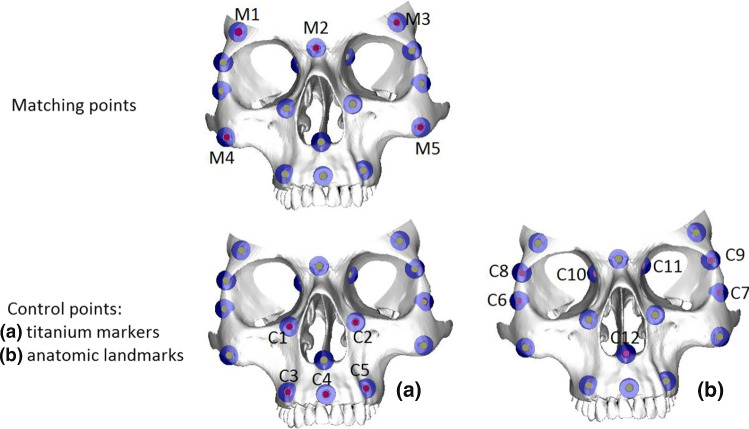
Location of fiducials for landmark-based registration and control points for TRE calculation

The tests were performed by two researchers on a physical phantom. The model was equipped with ten titanium markers. The coordinates of markers were measured manually on DICOM (on particular projections) and a STL bone model recorded by the 3D Artec Space Spider surface scanner. Five titanium markers (M1 to M5 in Fig. 6) were applied to calculate the landmark transform registration matrix (**LT**).

The DICOM data were transformed by the paired-points “Landmark Transform” registration matrix **LT,** and for the five titanium markers (M1 to M5) coordinates, Fiducial Registration Errors were calculated. Similarly, for five titanium markers (C1 to C5 in Fig. 6) and seven anatomical landmarks (C6 to C12 in Fig. 6) not used in registration calculation, the Target Registration Errors were computed. Both researchers performed measurements of all M1 to M5 and C1 to C12 points on DICOM and 3D Artec Space Spider scanner dataset twice for each of three resolutions. Results from two probes and two researchers were combined into three datasets (separately for specified resolution A 0.39 mm/pixel, B 0.43 mm/pixel, C 0.47 mm/pixel). For each set, the mean values of TRE were calculated from TRE obtained in selected control points.

In the second stage, the surface registration (**SR**) was performed.

For this algorithm, two uniform point clouds of equal resolution were created from both datasets. At the beginning, the user-defined HRPRs in selected regions of the facial part on the 3D Artec Space Spider scanner dataset, described results concern three defined HRPRs. The plane(s) determine uniform regular meshes of points with a defined resolution. The points were projected on the 3D Artec Space Spider scanner and DICOM dataset. As a result, two equally distributed high-resolution (containing 1324 points) datasets from projection on 3D Artec Space Spider scanner and DICOM dataset were obtained. Next, both clouds were registered by the surface registration method (Iterative Closest Point). The transformation matrix **SR** was calculated, and the DICOM dataset was transformed by it to match both datasets.

The main idea of registration is, of course, to provide the ability that surgeons can localize the navigated instrument on the image dataset. Therefore in the last stage, the 3D Artec Space Spider scanner dataset was registered with the data measured with a trackable pointer related to the **DRF** mounted on the skull phantom (navigation dataset). Before palpation with a navigated pointer, both researchers accomplished pivoting procedure (pivoting error was in range 0.11 to 0.20 mm). For the registration of both datasets to the **DRF** coordinate system, the Landmark Transform algorithm on five markers was applied. Both the 3D Artec Space Spider scanner and DICOM dataset were transformed by the **DRF** matrix obtained by registration. As a result, both 3D Artec Space Spider scanner and DICOM datasets were matched in the **DRF** coordinate system.

The result of the suggested **ERSR** algorithm was compared to the Landmark Transform Registration algorithm binding only the first and third phases of **ERSR** algorithm avoiding the surface registration.

To evaluate the result of registration, the colored maps of distance between the nearest points of the registered 3D Artec Space Spider scanner and DICOM datasets were calculated. The maps were generated both for **LTR** and **ERSR** algorithms.

In our research, the influence of DICOM resolution on the registration error was evaluated based on the histograms of distance maps. In the research, the following three resolutions were tested for a phantom with titanium markers: A (0.39 mm/pixel), B (0.43 mm/pixel), and C (0.47 mm/pixel).

## Results

The normal distribution of the study variables was verified using the Shapiro–Wilk test, while the results obtained were analyzed statistically using the Wilcoxon signed-rank test with alpha coefficient.

0.05. The first part of the analysis was to check the hypothesis that the errors of **ERSR** are similar to errors of landmark transform registration.

The analysis was performed for three resolutions of the DICOM dataset (A, B, C). During testing, two researchers were indicating points on DICOM and surface scanner dataset. The mean values of TRE were calculated from TRE in four control points (C6, C7, C9, C12).

Table 2 presents results of mean target registration error, standard deviations, and p-values of Wilcoxon signed-rank test.

**Table 2:** Averaged TRE (from 4 points) and p-value of Wilcoxon signed-rank test for LTR and ERSR on titanium markers (two researchers)

TRE for LTR [mm]	TRE for ERSR [mm]	*p*-value
resolution A		
1.2 ± 0.4	0.8 ± 0.3	0.008
resolution B		
1.3 ± 0.4	0.8 ± 0.5	0.000
resolution C		
1.5 ± 0.5	1.0 ± 0.7	0.002

To evaluate how much the last stage of registration (transformation to the **DRF** coordinate system) increases the error, we prepared Table 3. It presents the results of mean target registration errors before the last step transformation to the **DRF**, standard deviation, and p-values of Wilcoxon signed-rank test.

**Table 3:** TRE and p-value of the Wilcoxon signed-rank test for LTR and ERSR on titanium markers (two researchers) before transformation to DRF

TRE for LTR [mm]	TRE for ERSR [mm]	*p*-value
resolution A		
1.4 ± 0.4	0.7 ± 0.3	0.001
resolution B		
1.4 ± 0.3	0.7 ± 0.2	0.0002
resolution C		
1.4 ± 0.4	0.7 ± 0.3	0.001

**Fig. 7:**
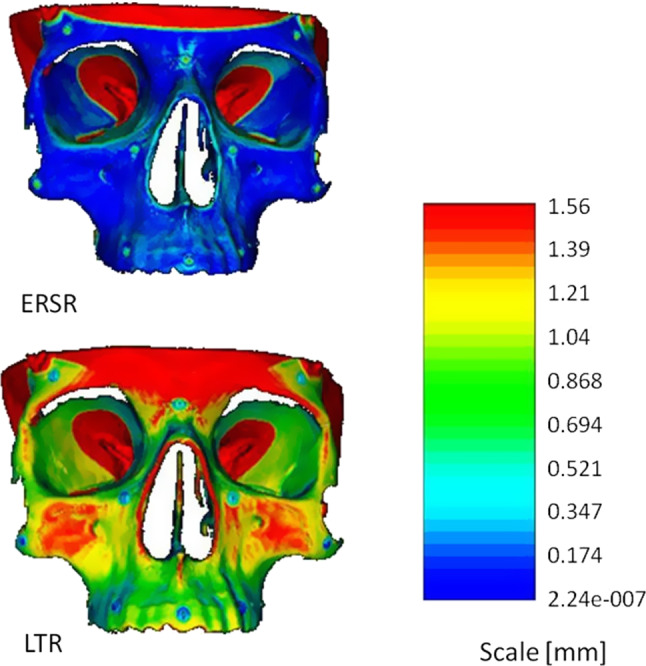
Exemplary map of distance for titanium markers, resolution A, upper image ERSR, lower image LTR (researcher B, probe2)

The second part of the analysis was to evaluate the map of distances between the model from scanner transformed to the **DRF** coordinate system and CT-based model transformed in the result of registration to the same coordinate system. The map presents the local distance between the given point and the nearest point of the second dataset. As a result, we obtained maps for both algorithms, three resolutions (A, B, C), and both researchers (each with two probes), for titanium markers. An exemplary result is shown in Fig. 7.

**Fig. 8:**
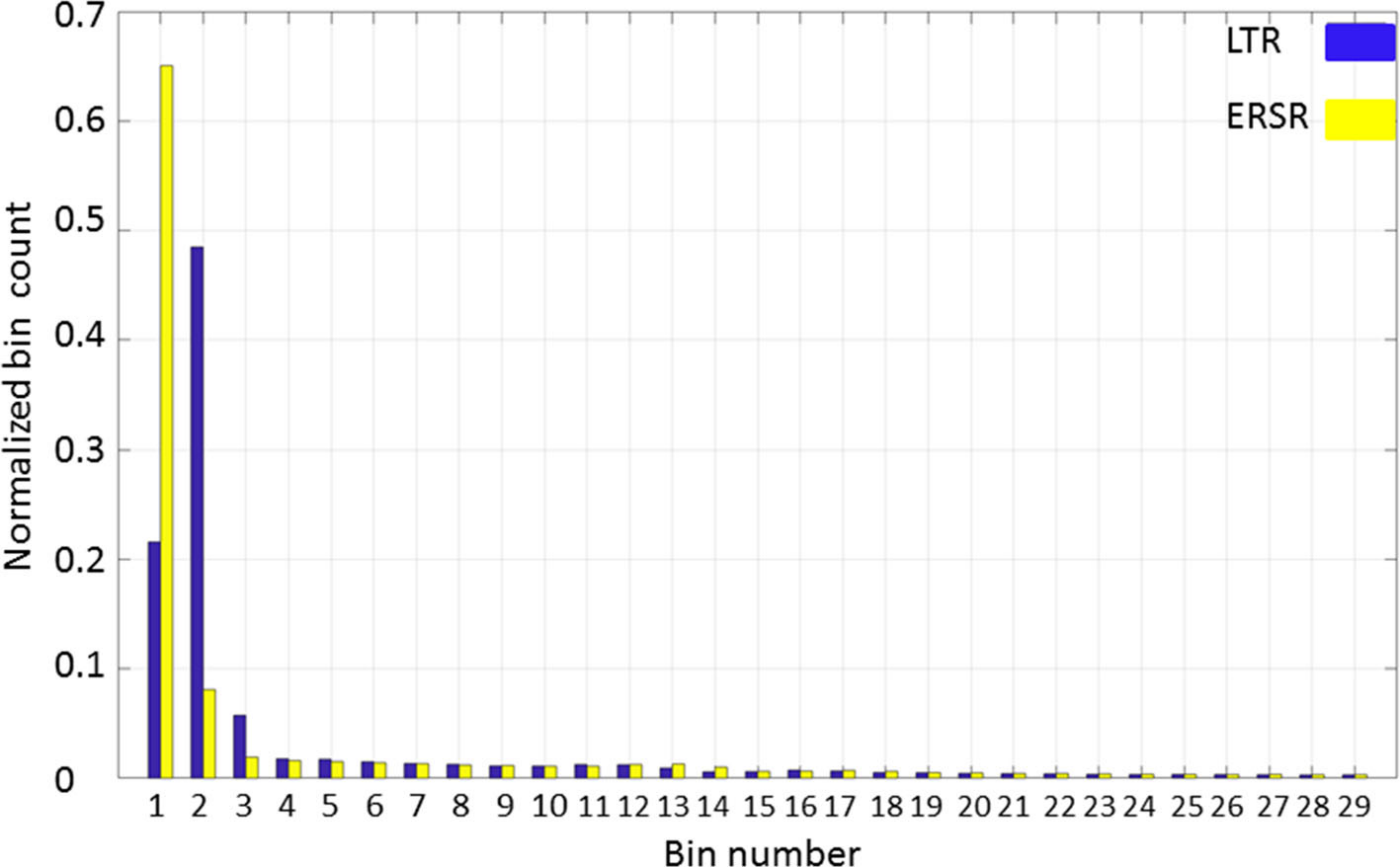
Exemplary histograms of map of distance for titanium markers, resolution A, yellow bins—ERSR, blue bins—LTR (researcher B, probe2)

To evaluate the maps, we calculated the histograms of maps of distances and analyzed the first bins for both algorithms (the lowest value of distances). The higher the frequency of the first bin, the better the outcome of registration for the whole model. The size of the bin depends on the highest value of distance in the map, for example in case of a maximal value 0.56 mm (as in Fig. 7), the bin size is about 0.05 mm. The exemplary histogram for the map of distance from Fig. 7 is shown in Fig. 8. In that case, the first bin covers a distance from 0 mm to 0.05 mm and the second bin from 0.051 to 0.1 mm.

Table 4 presents the first bin frequencies on the distance map in case of both methods of registration for titanium markers.

**Table 4:** First bin frequencies on histogram of distance map in case of both methods of registration for titanium markers

	LTR	ERSR
	resolution A	
Researcher A + B	0.21	0.64
	resolution B	
Researcher A + B	0.24	0.66
	resolution C	
Researcher A + B	0.22	0.64

## Discussion

The problem of registration methods accuracy is still present in scientific journals, especially between different modalities. Nowadays, the commercial systems usually offer only the registration with a trackable pointer, rarely a non-touching pointer or a form of scanner. A probable reason is an additional cost of the scanner (at least about 30 thousand USD), long time of acquisition (at least a few minutes), and long time of analyzing the dataset (depending on the PC processor and available memory even 20–30 min). Further development of registration methods is needed in order to reduce human factors, reduce the resulting error, simplify the procedure, shorten the time of acquisition and calculation, as well as increase matching efficiency.

The background for our investigation was the idea of applying a precise surface scanner. Our study aimed to check whether data from a precise surface scanner improves registration accuracy.

The presented work is a pilot study in which we focused on the analysis of a 3D printed skull phantom. As long as the bone itself is considered, there are no difficulties with scanner application also in humans. However, surface scanning of the skin would be prone to slight deformations related to the head positioning. For maximum accuracy, the patient position must be the same during CT scan and surface scanning during registration before computer-aided surgery. Furthermore, the tracheal tube during general anesthesia, and tools for head fixation, and the DRF must not interfere with the surface scan area. However, it is not necessary to use soft tissue scanning, since a limited exposed area of bone could serve as a stable region for registration using the ERSR method with projection.

In our approach on the bone phantom, we tested two algorithms of registration (Landmark Transform Registration **LTR** and developed Equal-Resolution Surface Registration algorithm **ERSR**), both applying data from the scanner.

Imprecise pointing of landmarks or markers both on DICOM/scanner and palpating them on the patient intraoperatively is critical for paired-points registration accuracy. Another factor is an incorrect segmentation of landmarks or markers on a DICOM-based 3D model. The surface registration stage helps to correct imprecise landmark transform matching involved by imprecise pointing and local errors of segmentation.

A crucial aspect is also the ability to reduce the influence of DICOM resolution on the result of registration. Even if the surface scanner dataset has a high resolution but the DICOM dataset has low resolution, the surface registration cannot work properly due to a lack of correspondence between points. We have proved that the HRPR is an answer to the problem. HRPR(s) helps to obtain a similar resolution of both datasets. Additionally, the user locates HRPR(s) to ensure the best matching in the region of interest (e.g., operation area).

An additional advantage of binding DICOM and scanning surface data is a visualization of bone and soft tissues, improving the limited resolution of DICOM bound with the scanner object.

Obtained averaged target registration errors and histogram analysis of distance maps revealed the significantly better capability to match datasets in case of equal-resolution surface registration algorithm for all resolution conditions. The differences are statistically significant. The frequency in the first bin of histogram of distance map is 3 times higher for the **ERSR** algorithm than in case of **LTR**.

We also analyzed the influence of applied types of markers on registration accuracy. It was observed that the TRE of **LTR** is lower than in the ICP case for titanium markers. The opposite relation is visible for anatomical markers. The reason is the problem of titanium marker segmentation. Markers appear to be protruding above the surface than they really are, and this causes high errors in the ICP method. However, the truly proper result is obtained in the ICP phase where the surfaces of tissues are matched. One must be aware that the result of registration expressed in the TRE value may be inconclusive. The accuracy of segmentation plays a crucial role in this process.

An important influence on the results of LT registration has the difficulty in pointing markers or anatomical landmarks on DICOM and scanner dataset, but the results of ERSR become less sensitive to that factor since the surface registration corrects the discrepancies. For that reason, in this pilot study we designed an experiment to be performed by two researchers.

We also analyzed the influence of the DICOM resolution and its impact on the registration error. Table 3 shows that the resolution of the DICOM dataset does not change the TRE in the **ERSR** method before registration to the **DRF**. The TRE is twice smaller than in the case of **LTR**, but in both cases, the TRE is stable with a change of resolution. The reason is probably the type of applied titanium markers, its small size, and easy interpretation in the DICOM dataset even if the resolution is worse. Table 2 shows that the resolution influences the accuracy of registration in the **LTR** method (the error increased with worse resolution 1.2 mm, 1.3 mm, 1.5 mm), but also in the worst case of resolution for the **ERSR** method (from 0.8 mm to 1.0 mm). But still, the value for ERSR is much lower than in the **LTR** case.

**Fig. 9:**
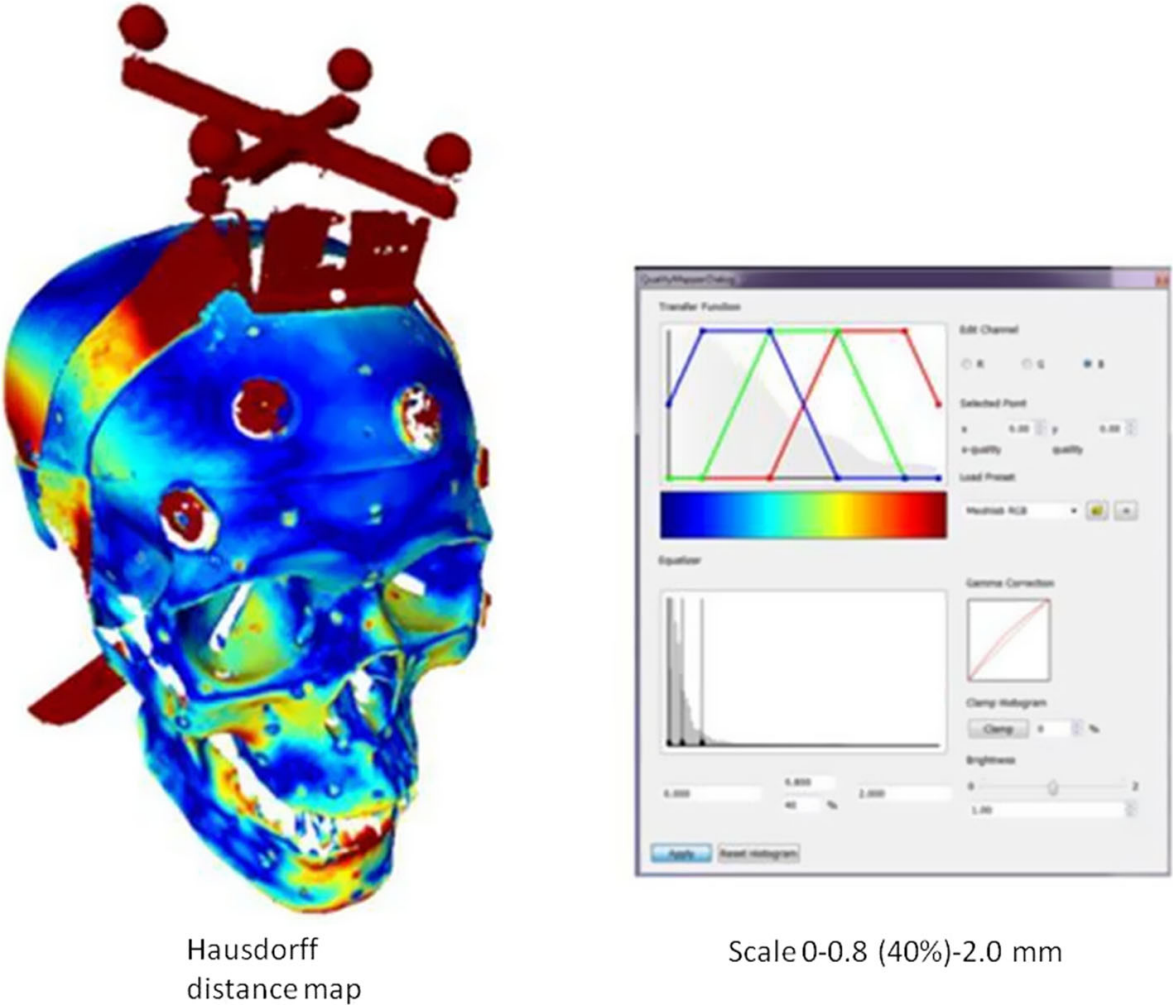
Hausdorff distance map between CT and scanner dataset, both transformed into the reference frame coordinate system based on recognized positions of spheres. The scale of distances 0- 0.8 (40%) − 2.0 mm

Comparing the results shown in Tables 2 and 3, we can observe an increase of TRE for the **ERSR** algorithm after the last stage—e.g., transformation by landmark transform calculated from data manually pointed on image/model and with a trackable pointer to the **DRF** coordinate system. To avoid that increase one could apply the approach proposed by Marmulla [19] or Fan [7]. Both groups of researchers suggested recognition of spherical markers and their centers in the scanner dataset and transformation of the dataset based on a definition of reference frame coordinate system regarding the positions of spheres. We checked that method. Our exemplary result of registration in the form of a Hausdorff distance map between CT and scanner dataset (both transformed into reference frame coordinate systems basing on recognized positions of spheres) is shown in Fig. 9. The highest influence on the registration error had the limited accuracy of the recognition of sphere markers.

A similar technique of registration based on surface scanning with markers, but without tracking by a navigation system, was also described by Fan [20]. Our results for the **ERSR** method are better than those obtained for two of three applied phantoms (our 0.7 mm versus 1.3 mm–1.5 mm), and a bit worse than the best result obtained in that work (our 0.7 mm versus 0.3 mm).

That analysis showed that adding the last stage of registration, e.g., paired-points registration to the **DRF** coordinate system did not worsen the resulting registration error more than obtained registration error in the technique of Marmulla [19] or Fan [20]. The disadvantage of the method is time-consuming complex scanning procedures to recognize all sphere markers (more than in case of scanning a specific bone area). A second deficiency is the need to scan the surface intraoperatively under time pressure conditions under anesthesia. We consider the scanning in the preoperative phase followed by **ERSR** of DICOM and scanner dataset as more ergonomic. The **LTR** approach in the last stage (transformation to DRF) is not ideal but a compromise between the time of intraoperative procedure and the precision.

Obtained results of the **ERSR** algorithm are comparable to those from the work of Choi [21] with data for the ICP algorithm recorded by a trackable pointer in relation to the dynamic reference frame. The advantage of our approach is the reliability that the scanner dataset is recorded on the tissue surface and the facility for data acquisition.

In our further studies, we will perform an analysis of reproducibility with a larger group of researchers and on various regions of projections. Moreover in our next study, we are also checking how the result of our study can influence aiding the surgery with augmented reality visualization [22, 23].
